# Development and validation of a CECT-based radiomics model for predicting IL1B expression and prognosis of head and neck squamous cell carcinoma

**DOI:** 10.3389/fonc.2023.1121485

**Published:** 2023-03-10

**Authors:** Yang Xie, Min Wang, Haibin Xia, Huifang Sun, Yi Yuan, Jun Jia, Liangwen Chen

**Affiliations:** ^1^ The State Key Laboratory Breeding Base of Basic Science of Stomatology, Hubei Province and Key Laboratory of Oral Biomedicine (Hubei-MOST and KLOBM), School and Hospital of Stomatology, Wuhan University, Wuhan, China; ^2^ Hubei-MOST and KLOBM, Department of Oral Implantology, School and Hospital of Stomatology, Wuhan University, Wuhan, China; ^3^ Department of Oral Radiology, School and Hospital of Stomatology, Wuhan University, Wuhan, China; ^4^ Department of Oral Maxillofacial-Head Neck Oncology, School and Hospital of Stomatology, Wuhan University, Wuhan, China

**Keywords:** head and neck squamous cell carcinoma, IL1B, contrast-enhanced computed tomography, radiomics model, prognosis

## Abstract

**Introduction:**

It is necessary to explore a noninvasive method to stratify head and neck squamous cell carcinoma (HNSCC)’s prognosis and to seek new indicators for individualized precision treatment. As a vital inflammatory cytokine, IL1B might drive a new tumor subtype that could be reflected in overall survival (OS) and predicted using the radiomics method.

**Methods:**

A total of 139 patients with RNA-Seq data from The Cancer Genome Atlas (TCGA) and matched CECT data from The Cancer Image Archive (TCIA) were included in the analysis. The prognostic value of IL1B expression in patients with HNSCC was analyzed using Kaplan-Meier analysis, Cox regression analysis and subgroup analysis. Furthermore, the molecular function of IL1B on HNSCC was explored using function enrichment and immunocytes infiltration analyses. Radiomic features were extracted with PyRadiomics and processed using max-relevance minredundancy, recursive feature elimination, and gradient boosting machine algorithm to construct aradiomics model for predicting IL1B expression. The area under the receiver operating characteristic curve (AUC), calibration curve, precision recall (PR) curve, and decision curve analysis (DCA) curve were used to examine the performance of the model.

**Results:**

Increased IL1B expression in patients with HNSCC indicated a poor prognosis (hazard ratio [HR] = 1.56, *P* = 0.003) and was harmful in patients who underwent radiotherapy (HR = 1.87, *P* = 0.007) or chemotherapy (HR = 2.514, *P* < 0.001). Shape_Sphericity, glszm_SmallAreaEmphasis, and firstorder_Kurtosis were included in the radiomics model (AUC: training cohort, 0.861; validation cohort, 0.703). The calibration curves, PR curves and DCA showed good diagnostic effect of the model. The rad-score was close related to IL1B (*P* = 4.490*10-9), and shared the same corelated trend to EMT-related genes with IL1B. A higher rad-score was associated with worse overall survival (*P* = 0.041).

**Discussion:**

The CECT-based radiomics model provides preoperative IL1B expression predictionand offers non-invasive instructions for the prognosis and individualized treatment of patients withHNSCC.

## 1 Introduction

Head and neck squamous cell carcinoma (HNSCC) is the sixth most common cancer worldwide and covers a variety of epithelial malignancies that develop in the tongue, oral cavity, nasal cavity, jaw, pharynx, and larynx (1). Patients with HNSCC have poor outcomes owing to the high rates of recurrence and metastasis, with a 5-year survival rate of 40%-50% (2). From a clinical perspective, predictive and prognostic detection methods play a pivotal role in treatment, precision medicine, and prediction of overall outcomes. Commonly used prognostic indicators of HNSCC include tumor-node-metastasis (TNM) staging (3), human papilloma virus (HPV) (4), and immune infiltration (5, 6). Most prognostic indicators are obtained using invasive methods. Thus, expand the search beyond the TNM staging system to explore novel, non-invasive prognostic method is necessary to stratify patient prognosis and to provide new indicators for individualized precision treatment.

The inflammatory response significantly influences tumor growth and its response to clinical treatment (7). Interleukin 1 beta (IL1B), an important cytokine in the inflammatory response and a vital mediator of cell pyroptosis and apoptosis (8), has been actively investigated in various types of squamous carcinomas, including lung squamous cell carcinoma (9), cutaneous squamous cell carcinoma (10), and esophageal carcinoma (11), but especially oral squamous cell carcinoma (12). IL1B is an excellent indicator of cellular radioresistance and senescence in HPV-negative HNSCC cells without functional involvement in these processes (13). Macrophage secretory IL1B promotes docetaxel resistance in HNSCC (14). Increased IL1B expression and secretion by cancer stem cells may activate an autocrine signaling loop that promotes epithelial-mesenchymal transition (15, 16), cellular invasion (17) and distant colonization by stimulating NF-κB and CREB signaling pathways (18). Moreover, canakinumab, an IL1B inhibitory antibody, significantly decreased the incidence and mortality of lung cancer (19, 20). Canakinumab used before surgery can improve the success rate of surgery and may help prevent cancer recurrence (21). However, no effective and non-invasive methods to detect IL1B expression in HNSCC tumor currently exist, other than surgical procedures combined with pathological examinations.

Radiomics is a conventional noninvasive strategy for detecting HNSCC. As a non-invasive method, contrast-enhanced computed tomography (CECT) can detect soft tissue and bone involvement, whereas magnetic resonance imaging is primarily used to detect soft tissue lesions. Radiomics is a newly developed technology that can quantify tumor characteristics on the basis of massive radiomic image data, which can non-invasively help assess tumor phenotype and heterogeneity (22) and provide dynamically detected and quantitatively determined details on tumor biology and pathophysiology (23). The radiomics approach has been applied for the diagnostic, therapeutic, and prognostic evaluation of a variety of cancers (24). CT-based radiomics analysis has been shown to predict preoperative HPV status (25) and pathologic grade (26) of HNSCC. However, no reports exist on predicting IL1B expression in HNSCC tumors and patients’ overall survival (OS) time on the basis of a radiomics imaging model.

In this study, we explored the molecular function of IL1B in HNSCC on the basis of bioinformatic analysis; developed a CECT-based radiomics model using maximum-relevance minimum-redundancy (mRMR), recursive feature elimination (RFE), and gradient boosting machine (GBM) method; and evaluated the clinical utility of the radiomics model for non-invasive preoperative prediction of IL1B expression in HNSCC and prognosis of patients with HNSCC.

## 2 Materials and methods

### 2.1 Molecular function of IL1B in HNSCC

#### 2.1.1 Data source

**Figure 1:**
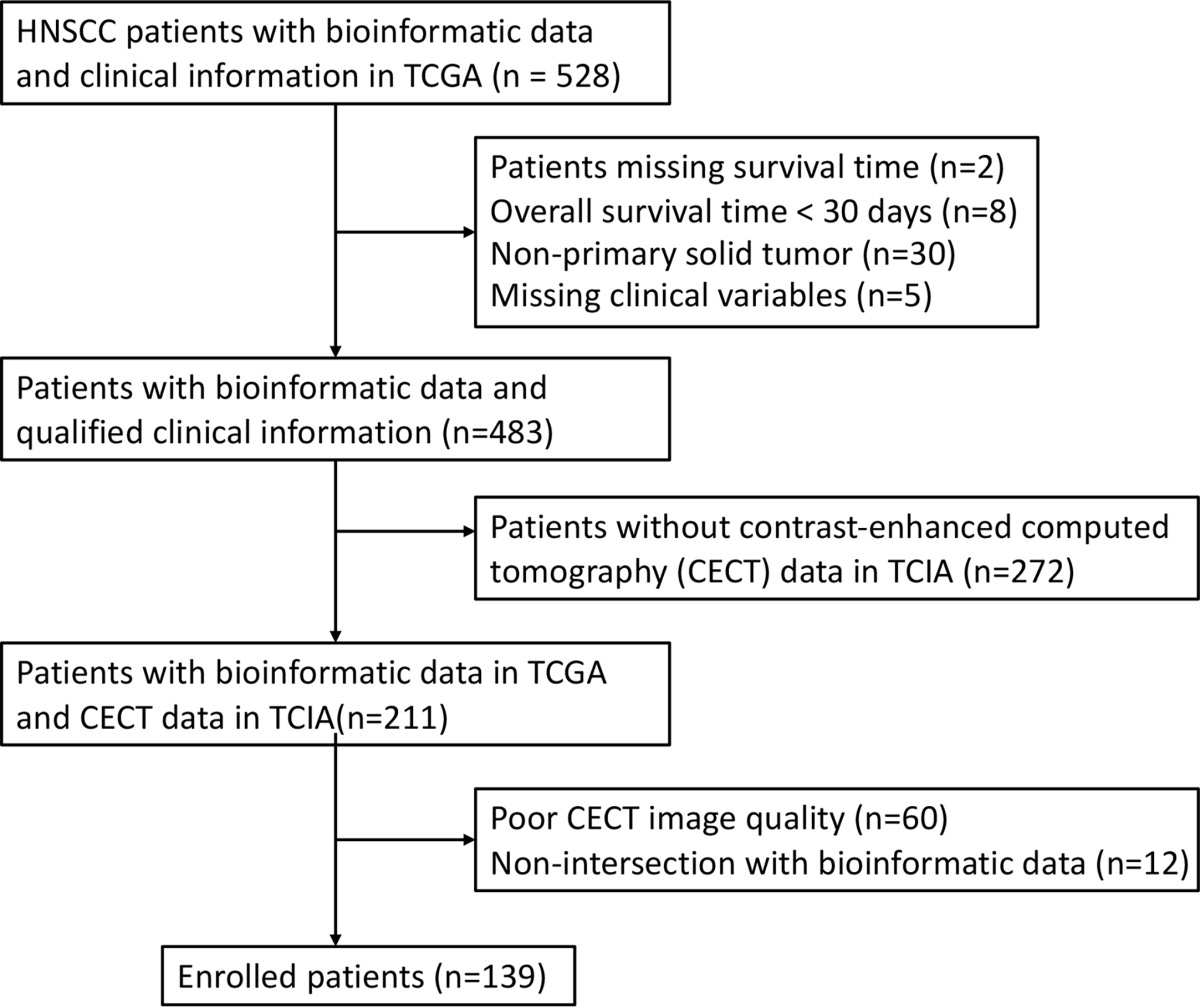
The Inclusion and Exclusion Procedure of the patients with HNSCC.

In this study, the RNA-seq data and clinical information of 528 patients with HNSCC were obtained from The Cancer Genome Atlas (TCGA). A total of 45 patients were excluded owing to missing survival time (n = 2), OS time < 30 days (n = 8), non-primary solid tumor (n = 30) or missing clinical variables (n = 5). Among the remaining 483 patients, CECT data of 211 patients were obtained within 2 weeks before surgery and corresponding clinical information were obtained from The Cancer Imaging Archive (TCIA). A total of 72 patients were excluded because of poor image quality (n = 60) and non-intersection with bioinformatic data (n = 12). After exclusion, 139 patients were enrolled in the study (**Figure 1**). Genomic data for HNSCC and normal tissues were obtained from the University of California Santa Cruz Xena platform.

#### 2.1.2 Kaplan–Meier analysis

Kaplan–Meier (KM) plotting analysis was used to evaluate the prognostic value of IL1B in HNSCC using the “survival” and “survminer” packages in R (version 4.0.4). The cut-off value of IL1B expression in HNSCC tumor tissues was determined as 3.383 using the surv_cutpoint function of the “survminer” package in R. Accordingly, 483 patients were separated into two groups: those with high IL1B expression (n = 191) and those with low IL1B expression (n = 292). The log-rank test was used to estimate the survival probability, median survival times, and 95% confidence intervals (CIs) of the two groups. Because the KM curves of the IL1B high and low expression groups crossed, conditional landmark KM analyses of survival percentage were performed at 12, 24, 36, 48, 60, 72, 84 and 96 months to compare the survival curves stratified by IL1B expression. Landmark KM curves were plotted using the “jskm” package.

#### 2.1.3 Cox regression analyses and subgroup analysis

Through univariate Cox regression analysis, the prognostic value of the clinical and pathological features of HNSCC was identified. Since the listed features were proven to be common and important variables associated with OS in the clinic and in the literature, they were all included in the multivariate Cox analyses. Cox regression analyses were conducted with “survival” and “forestplot” packages in R.

Subsequently, subgroup analysis was utilized using “cmprsk”, “survival” and “forestplot” packages in R to evaluate the effect of IL1B in each subgroup. Interactions between IL1B and prespecified subgroups were evaluated by including age, gender, radiotherapy, chemotherapy, primary tumor site, HPV status, perineural invasion, T stage, N stage, M stage, and tumor grade in the Cox regression model.

#### 2.1.4 Exploration of IL1B function in HNSCC

By using the “clusterProfiler” package in R, we analyzed the enriched terms in the Gene ontology (GO) and the Kyoto Encyclopedia of Genes and Genomes (KEGG) to get insight into the IL1B-related pathways. Differentially expressed genes were obtained using the “limma” package in R, with the threshold set at |log2FC| >1 and adj*P* < 0.05, by comparing the high and low IL1B expression groups. We employed the molecular function (MF), biological process (BP), and cellular component (CC) annotation databases for GO enrichment. The lollipop figures show the top 30 GO terms and top 30 KEGG pathways. Furthermore, 483 RNA-seq data were uploaded to the CIBERSORTx database to characterize the immune cell composition (27). Spearman’s rank correlation analysis was performed to examine the relationship between IL1B expression and immunocyte infiltration.

### 2.2 Development and validation of a CECT-based radiomics model for IL1B

#### 2.2.1 Region of interest segmentation and image feature extraction

A professional radiologist performed region of interest (ROI) segmentation on all 139 CECT interpretations using the Medical Imaging Interaction Toolkit without referring to any clinical information. To avoid artificial and subjective deviations, another radiologist randomly selected 20 patients for the test and retest analysis and multiclinician segmentation. The radiomics features retrieved from the ROI segmentation were automatically extracted using PyRadiomics, an open-source program with 107 radiomics features, to assess the first-order statistical, morphological, and texture features. **Table S3** displays all the radiomic feature categories. Prior to feature extraction, the data were standardized using Z-score normalization. The intraclass correlation coefficient (ICC) was used to measure the repeatability of both radiologists delineating the ROI. ICC > 0.8 represents high consistency, 0.51-0.79 represents medium consistency, and < 0.5 represents poor consistency. ICC < 0.8 features were omitted from further analysis.

#### 2.2.2 Radiomics feature selection

A total of 139 patients were randomly assigned to the training and validation cohorts in a ratio of 6:4. The mRMR method was used to prioritize feature significance to reduce overfitting of the data and locate the best linked features. The mutual information (MI) to class labels was maximized, and MI with other characteristics was minimized to rank the input features. The RFE method was then applied to the top 20 features for high IL1B expression in the training cohort. During each iteration, the feature that contributed the least to enhancing the performance of the model was eliminated from the selection process. Feature ranking was determined using the number of iterations in which the feature was eliminated.

#### 2.2.3 Radiomics model construction

The features were then centered and scaled using the caret preprocess function and analyzed using the GBM method, which is a machine learning approach suitable for regression and classification issues. The “gbm” function in the “caret” package in R was used to produce a projected class probability score and construct a GBM model. Additionally, we employed a leave-one-out-based cross-validation approach to train and test the GBM model to minimize bias in feature selection. The GBM model was trained using all other samples in the dataset and was evaluated on the remaining sample that was not part of the training cycle. The top performing model was maintained and used. For each sample, the model generated a radiomics score (rad-score). The expression of IL1B was higher when the rad-score was higher.

#### 2.2.4 Radiomics model evaluation

The radiomics model was developed from the training cohort and estimated in the validation cohort. Receiver operating characteristic (ROC) curves were used to evaluate the diagnostic ability of the model. The evaluation indicators included the area under the ROC curve (AUC), accuracy (ACC), and specificity (SPEC). The validation cohort was independently validated to evaluate prediction accuracy. The calibration degree of the radiomics model was examined by performing the Hosmer–Lemeshow goodness-of-fit test and drawing the calibration curve. To evaluate whether the model is affected by data imbalances, the precision recall (PR) curve was generated using the “modEvA”package in R. To evaluate clinical utility, decision curve analysis (DCA) was performed using the "rmda" package in R. The Delong test was used to compare the diagnostic efficiency of each model.

### 2.3 Clinical utility

#### 2.3.1 Correlation between rad-score and IL1B expression

The rad-score for each patient was computed using a radiomics model. Rad-scores were compared between the IL1B high- and low-expression group in the training and validation cohorts using the Wilcoxon test. A recent study demonstrated that IL1B pro-motes tumor metastatic outgrowth by affecting the epithelial-to-mesenchymal transition (EMT) of tumor cells (28). In this study, we focused on the potential association among rad-scores, IL1B expression, and EMT-related gene expression using Spearman correlation analysis with the “stats” package in R.

#### 2.3.2 Survival analysis using radiomics model

The “survminer” package in R was used to assess the rad-score and OS time of the 139 patients involved in the research. Using the greatest Youden index, the cut-off value for the rad-score was found to be 0.445. Accordingly, the patients were separated into two groups: those with high rad-scores (n = 69) and those with low rad-scores (n = 70). The KM curve was utilized to calculate survival probability and median survival time. The log-rank test was used to compare the survival probabilities of patients in the two groups.

#### 2.3.3 Statistical analyses

SPSS (version 25.0) and R software (version 4.0.4) were used for all statistical analyses. Continuous and categorical variables are shown as medians. The optimal cut-off value of IL1B expression in HNSCC with continuous values was determined using the “survminer” package. The cut-off value of the rad-score was identified as the highest Youden index of the ROC curve. Categorical variables were compared using the Chi-square test or Fisher’s exact test. Continuous variables were compared utilizing the t-test or Mann–Whitney U-test. Statistical significance was defined as a two-tailed *P-value* < 0.05.

## 3 Results

### 3.1 Molecular function of IL1B on HNSCC

#### 3.1.1 Characteristics of the study population

Four hundred and eighty-three patients with HNSCC from the TCGA database were included in this study. The cut-off value of IL1B expression was determined as 3.383 using the “survminer” package in R. Accordingly, the patients were separated into two groups: those with high IL1B expression (n = 191, IL1B expression > 3.383) and those with low IL1B expression (n = 292, IL1B expression < 3.383). **Table 1** shows the baseline patient characteristics. There were no statistically significant differences in age, gender, chemotherapy, primary tumor site, perineural invasion, T stage, N stage, M stage, and tumor grade between the IL1B high- and low-expression groups (*P* > 0.05), except for HPV status (*P* = 0.012) and radiotherapy (*P* = 0.04).

**Table 1:** Baseline characteristics of patients with HNSCC in the IL1B high- and low-expression group.

Variables	Total (n=483)	Low expression (n=293)	High expression (n=191)	*P* ^a^
**Age, n (%)**				0.716
~59	211 (44)	130 (45)	81 (42)	
60~	272 (56)	162 (55)	110 (58)	
**Gender, n (%)**				0.147
Female	128 (27)	70 (24)	58 (30)	
Male	355 (73)	222 (76)	133 (70)	
**Radiotherapy, n (%)**				0.04 *
No	234 (48)	153 (52)	81 (42)	
Yes	249 (52)	139 (48)	110 (58)	
**Chemotherapy, n (%)**				0.717
No	322 (67)	197 (67)	125 (65)	
Yes	161 (33)	95 (33)	66 (35)	
**Primary tumor site, n (%)**				0.086
Larynx	109 (23)	66 (23)	43 (23)	
Oral Cavity	297 (61)	171 (59)	126 (66)	
Oropharynx/Hypopharynx	77 (16)	55 (19)	22 (12)	
**HPV status, n (%)**				
Negative	68 (14)	35 (12)	33 (17)	0.012 *
Positive	30 (6)	25 (9)	5 (3)	
Unkown	385 (80)	232 (79)	153 (80)	
**Perineural invasion, n (%)**				0.77
No	181 (37)	110 (38)	71 (37)	
Yes	161 (33)	94 (32)	67 (35)	
Unknow	141 (29)	88 (30)	53 (28)	
**T stage, n (%)**				0.685
T1/T2	173 (36)	102 (35)	71 (37)	
T3/T4/Tx/Unkown	310 (64)	190 (65)	120 (63)	
**N stage, n (%)**				0.267
N0	164 (34)	93 (32)	71 (37)	
N1/N2/N3/Nx/Unknown	319 (66)	199 (68)	120 (63)	
**M stage, n (%)**				0.06
M0	174 (36)	95 (33)	79 (41)	
M1/M2/Unknow	309 (64)	197 (67)	112 (59)	
**Grade, n (%)**				0.153
G1/G2	348 (72)	203 (70)	145 (76)	
G3/G4/Gx	135 (28)	89 (30)	46 (24)	

a P value from chi-square test. * P < 0.05.

#### 3.1.2 KM analysis

**Figure 2:**
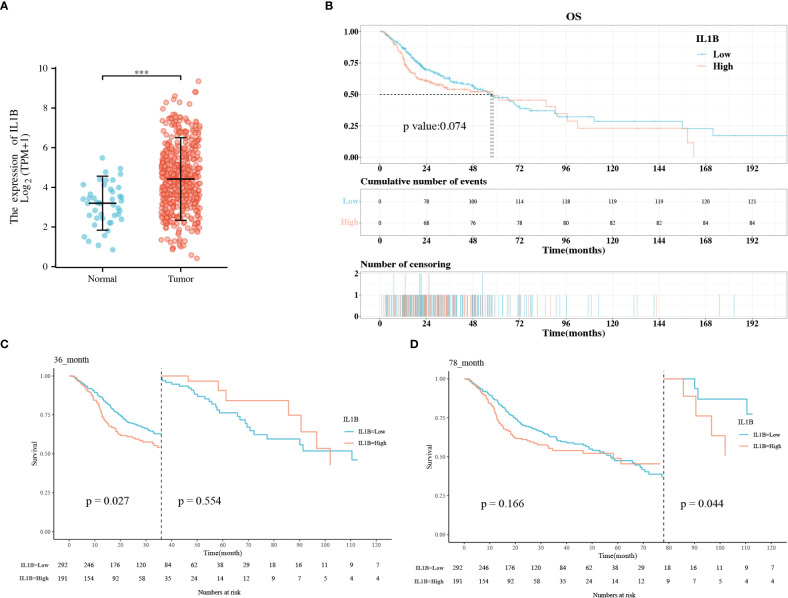
The correlation analysis between IL1B expression level and OS of patients with HNSCC. **(A)** The expression level of IL1B in HNSCC group and normal group; **(B)** The KM analysis of IL1B high- and low-expression groups; **(C, D)** The landmark analysis of IL1B high- and low-expression group. OS, overall survival; KM, Kaplan–Meier.

IL1B was significantly overexpressed in HNSCC tissues than in healthy tissues (*P* < 0.001), and the median difference between the two groups was 1.338 (**Figure 2A**). According to the KM survival curves, there was a statistical critical level of difference between the IL1B high- and low-expression groups (*P* = 0.074, **Figure 2B**). Owing to the crossovers on the KM curve, we conducted a landmark KM analysis. In the 0-36 months, the IL1B low expression group had a considerably better survival rate than the IL1B high expression group (*P* = 0.027, **Figure 2C**). The survival rate from 48-78 months was not significantly different between the groups (*P* > 0.05). From 78-120 months in the late stage, the survival rate was higher in the IL1B low expression group (*P* = 0.044, **Figure 2D**). In conclusion, survival analysis shows that high IL1B expression in patients with HNSCC in early (< 36 months) and late stages (> 78 months) indicates a poor survival probability.

#### 3.1.3 Cox regression and subgroup analyses

**Figure 3:**
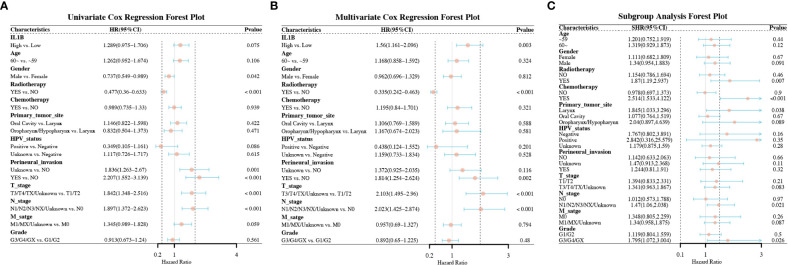
Forest plots of the factors correlated with the OS of patients with HNSCC. **(A)** The forest plot of univariate Cox regression analysis; **(B)** The forest plot of multivariate Cox regression analysis; **(C)** The forest plot of subgroup analysis. OS, overall survival.

We found that high IL1B expression was a risk factor for OS in univariate Cox analysis with a critical level of statistical significance (High vs. Low) (HR = 1.289, 95% CI:0.975-1.706, *P* = 0.075, **Figure 3A**). After multivariate adjustment, high IL1B proved to be a risk factor for OS (High vs. Low) (HR = 1.56, 95%CI:1.161-2.096, *P* = 0.003, **Figure 3B**). “HR = 1.56” indicates that high IL1B expression significantly increased the risk of death in patients with HNSCC. Differentially expressed IL1B exhibited a prognostic value in some clinicopathological subgroups (**Figure 3C**), including patients underwent radiotherapy (High vs. Low) (HR = 1.87, 95% CI: 1.19-2.937, *P* = 0.007), patients underwent chemotherapy (High vs. Low) (HR = 2.514, 95% CI: 1.533-4.122, *P* < 0.001), primary tumor in the larynx (High vs. Low) (HR = 1.845, 95% CI: 1.033-3.296, P = 0.038), tumors in N1/N2/N3/NX stages (High vs. Low) (HR = 1.47, 95% CI: 1.06-2.038, *P* = 0.021), and tumors in GE/G4/GX grade (High vs. Low) (HR = 1.795, 95% CI: 1.072-3.004, *P* = 0.026). The above analyses indicate that increased IL1B expression in patients with HNSCC predicts a potentially poor prognosis.

#### 3.1.4 Exploration of the IL1B function in HNSCC

**Figure 4:**
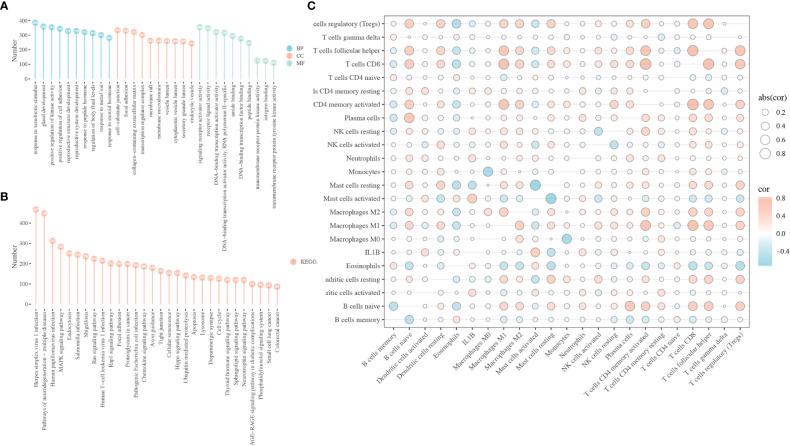
Biological function of IL1B in HNSCC. **(A)** GO enrichment analysis of the DEGs between the IL1B high- and low-expression group, including BP, CC, and MF categories. **(B)** KEGG analysis of the DEGs between the IL1B high- and low-expression group. **(C)** The immunocytes infiltration and IL1B expression level in HNSCC tissues. GO, Gene oncology; KEGG, Kyoto Encyclopedia of Genes and Genomes; BP, biological process; CC, cellular component; MF, molecular function.

To investigate the potential mechanism underlying the role of IL1B in HNSCC, functional enrichment analysis was performed on DEGs between the IL1B high- and low-expression groups using the “clusterProfile” package in R. In the BP, CC, and MF categories, the most significantly enriched GO terms relative to the IL1B expression groups were response to xenobiotic stimulus, cell-substrate junction, and signaling receptor activator activity, respectively (**Figure 4A**). The most enriched KEGG pathways were herpes simplex virus 1 infection, pathways of neurodegeneration-multiple diseases, HPV infection, and MAPK signaling pathway, et al. (**Figure 4B**). Owing to the significance of IL1B, the association between immunocyte infiltration and IL1B expression was further investigated using the CIBERSORTx website, as shown in **Figure 4C**.

### 3.2 Development and validation of a CECT-based radiomics model for IL1B

#### 3.2.1 Intraclass correlation coefficient and feature extraction

**Figure 5:**
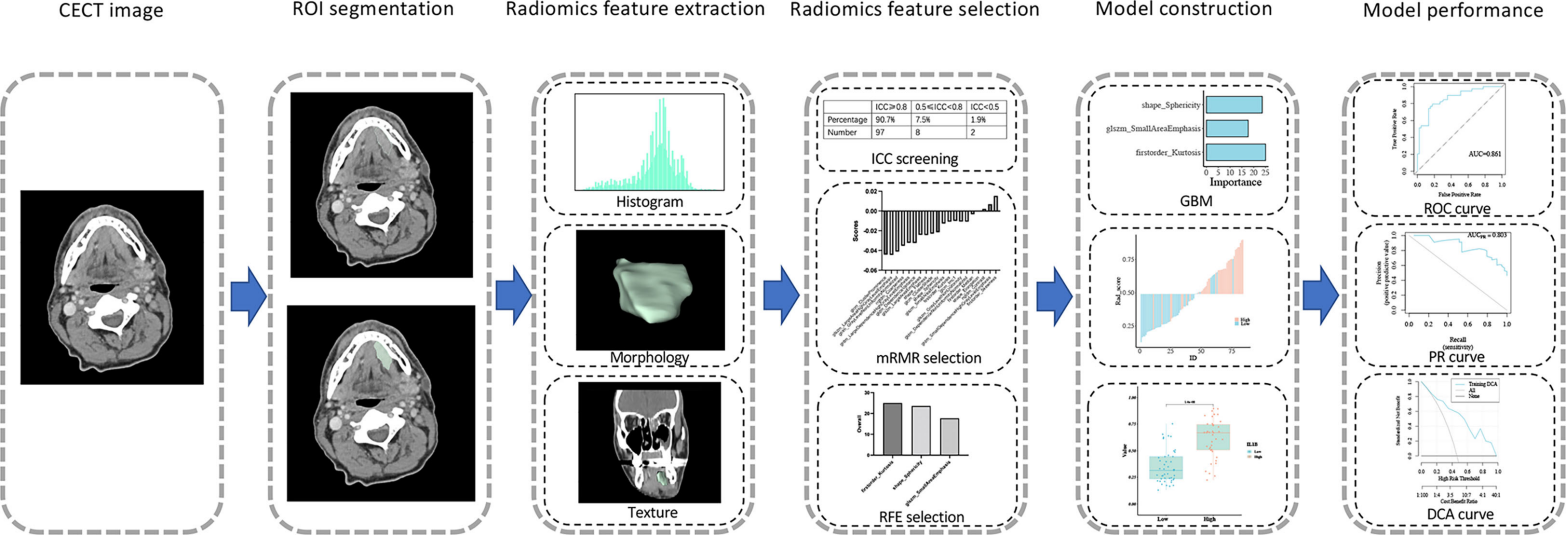
Workflow of construction and evaluation of the radiomics model.

Among the 483 patients with RNA-Seq data in TCGA, 139 patients with corresponding CECT data were acquired from TCIA and used to develop a radiomics model for IL1B expression. The workflow is shown in **Figure 5**. Two professional radiologists extracted 107 imaging features for each patient. The reproducibility of the radiomics features was assessed using the ICC (**Table S1**). The mean ICC value of the imaging features was 0.941, and 97 radiomics features with an ICC value ≥ 0.80 (90.7% of all features) were included in in the follow-up feature selection.

#### 3.2.2 Feature selection and radiomics model construction

A total of 139 patients were randomly assigned to the training cohort (n = 84) and validation cohort (n = 55) at a 6:4 ratio. Regarding clinical characteristics and IL1B expression, no significant differences were observed between the training and validation cohorts (*P* > 0.05 for all, **Table S2**). All 97 extracted features (ICC ≥ 0.80) were included in the mRMR algorithm, which selected 20 optimal features for further analysis (**Table S3**). Then, the 20 features were imported into the RFE algorithm, and the top three features were adopted as the best features. The three features were shape_Sphericity, glszm_SmallAreaEmphasis, and firstorder_Kurtosis.

**Figure 6:**
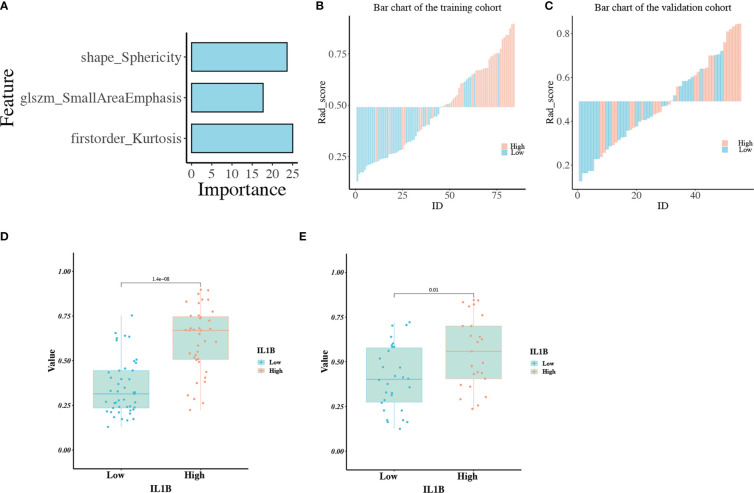
**(A)** The importance of each feature in the radiomics model; **(B, C)** The performance for IL1B expression prediction of the radiomics model in the training and validation cohorts; **(D, E)** The comparison of rad-scores between IL1B high- and low-expression groups in the training and validation cohorts.

We trained these three features using the GBM algorithm and the leave-one-out-based cross-validation approach to obtain an ideal machine learning model for predicting IL1B expression in HNSCC tissues. **Figure 6A** outlines the importance of each feature in the final radiomics model (**Table S4**). **Figures 6B, C** depicts the performance of the IL1B expression prediction. In the training cohort, the rad-score of the radiomics model was substantially greater in the group with high IL1B expression than that in the group with low IL1B expression (*P* = 1.404*10^-8^, **Figure 6D**). This trend was further confirmed in the validation cohort (*P* = 0.01; **Figure 6E**).

#### 3.2.3 Radiomics model performance

**Figure 7:**
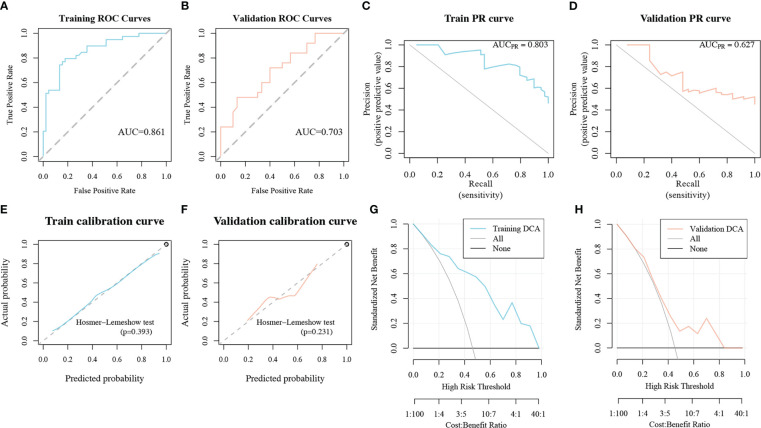
The performance of the radiomics model in the IL1B grading evaluation. **(A, B)** ROC curves of the radiomics model in training and validation cohorts. **(C, D)** PR curves of the radiomics model in training and validation cohorts. **(E, F)** Calibration curves of the radiomics model in training and validation cohorts. **(G, H)** DCA curves of the radiomics model in training and validation cohorts. ROC, Receiver operating characteristic; PR, precision recall; DCA, decision curve analysis.

The radiomics model performed well in the IL1B grading evaluation based on ROC curve analysis. The AUC of the radiomics model for the training group was 0.861 (95%CI:0.782-0.94, **Figure 7A**), with ACC: 0.81, SPEC: 0.822. The AUC of the radiomics signature for the validation group was 0.703 ((95%CI:0.565-0.842, **Figure 7B**), with ACC: 0.618 and SPEC: 0.667. The AUC of the PR curve in the training cohort was 0.803 (**Figure 7C**), whereas the AUC of the PR curve in the validation cohort was 0.627 (**Figure 7D**). The *P*-values of the H-L goodness of fit test were greater than 0.05 (training cohort: *P* = 0.393, **Figure 7E**; validation cohort: *P* = 0.231, **Figure 7F**), indicating a good agreement between the evaluated IL1B expression by the calibration curve of the model and the actual IL1B expression. **Figure 6** depicts the radiomics model’s DCA curve. The DCA in the training cohort revealed that if patients’ high-risk threshold is between 15%-100%, utilizing the radiomics model to forecast IL1B expression offers greater value than “ALL” and “NONE” strategies (**Figure 7G**). The DCA curves for the validation cohort also demonstrated that the radiomics model adds greater benefit than “ALL” and “NONE” techniques in the 30%-85% high-risk threshold (**Figure 7H**), indicating high clinical utility. The Delong test demonstrated that the AUCs of the training cohort were not markedly different from those of the validation cohort (*P* > 0.05) in this study.

### 3.3 Clinical utility

#### 3.3.1 Correlation analysis with epithelial-mesenchymal transition

**Figure 8:**
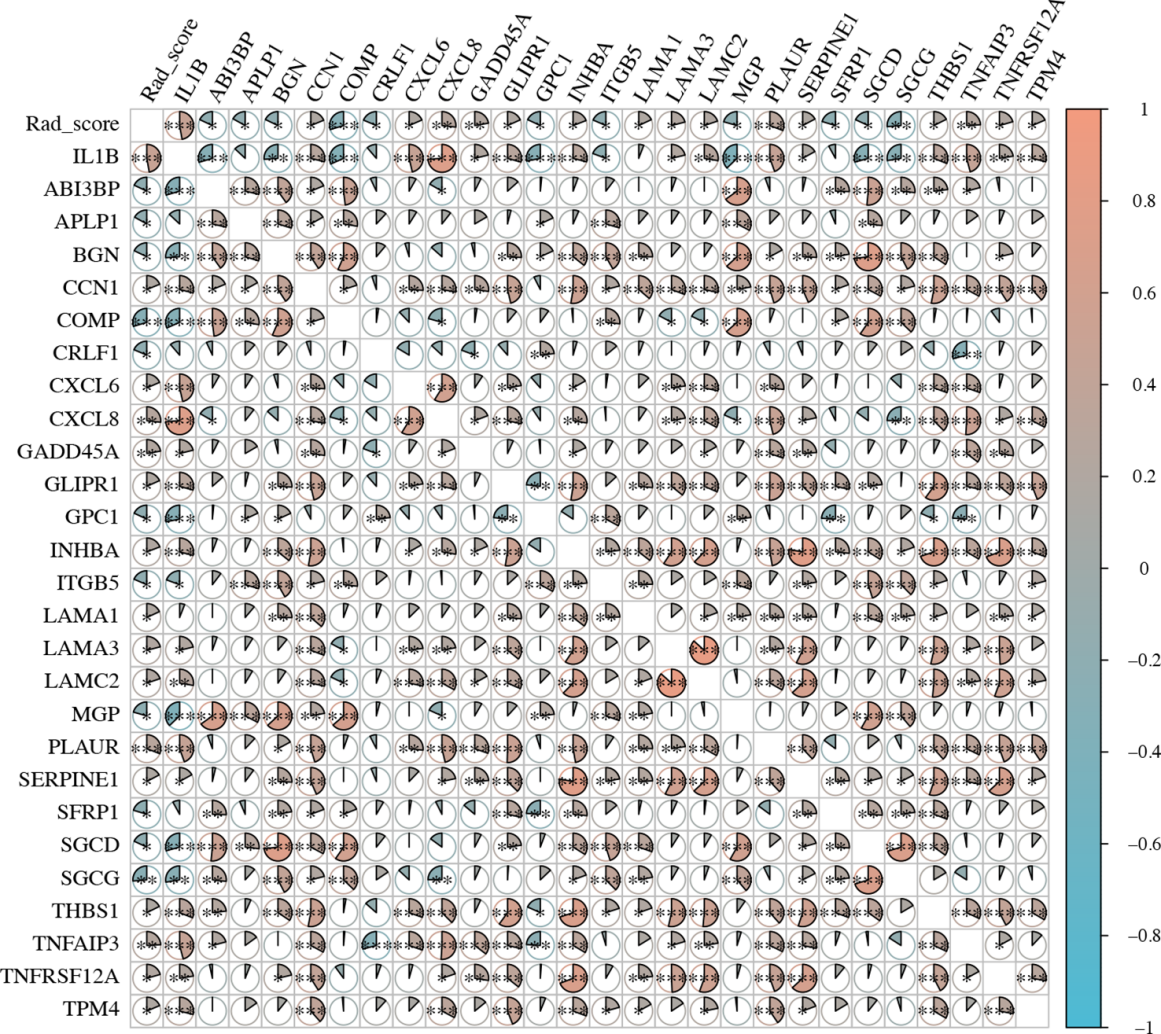
The expression level of EMT-related genes and rad-scores in HNSCC. EMT, Epithelial-Mesenchymal Transition; HNSCC, Head and neck squamous cell carcinoma.

Because EMT is a common cellular phenotype that occurs only in tumors, we further explored the correlation among rad-score, IL1B expression, and EMT-related genes (**Table S5**) expression in HNSCC tissue. The rad-score was significantly positively correlated with IL1B expression (cor = 0.472, *P* = 4.490*10^-9^). The correlation between the rad-score and EMT-related genes was similar to that between IL1B expression and EMT-related genes. IL1B expression was positively and significantly correlated with CNN1, CXCL6, CXCL8, GADD45A, GLIPR1, INHBA, LAMA3, LAMC2, PLAUR, SERPINE1, THBS1, TNFAIP3, TNFRSF12A, and TPM4 (*P* < 0.05), and the rad-score was also positively and significantly correlated with these genes. IL1B expression was negatively and significantly correlated with ABI3BP, BGN, COMP, GPC1, ITGB5, MGP, SGCD, and SGCG (*P* < 0.05), whereas the rad-score had a similar relationship with EMT-related genes (**Figure 8**).

#### 3.3.2 Survival analysis using the radiomics model

**Figure 9:**
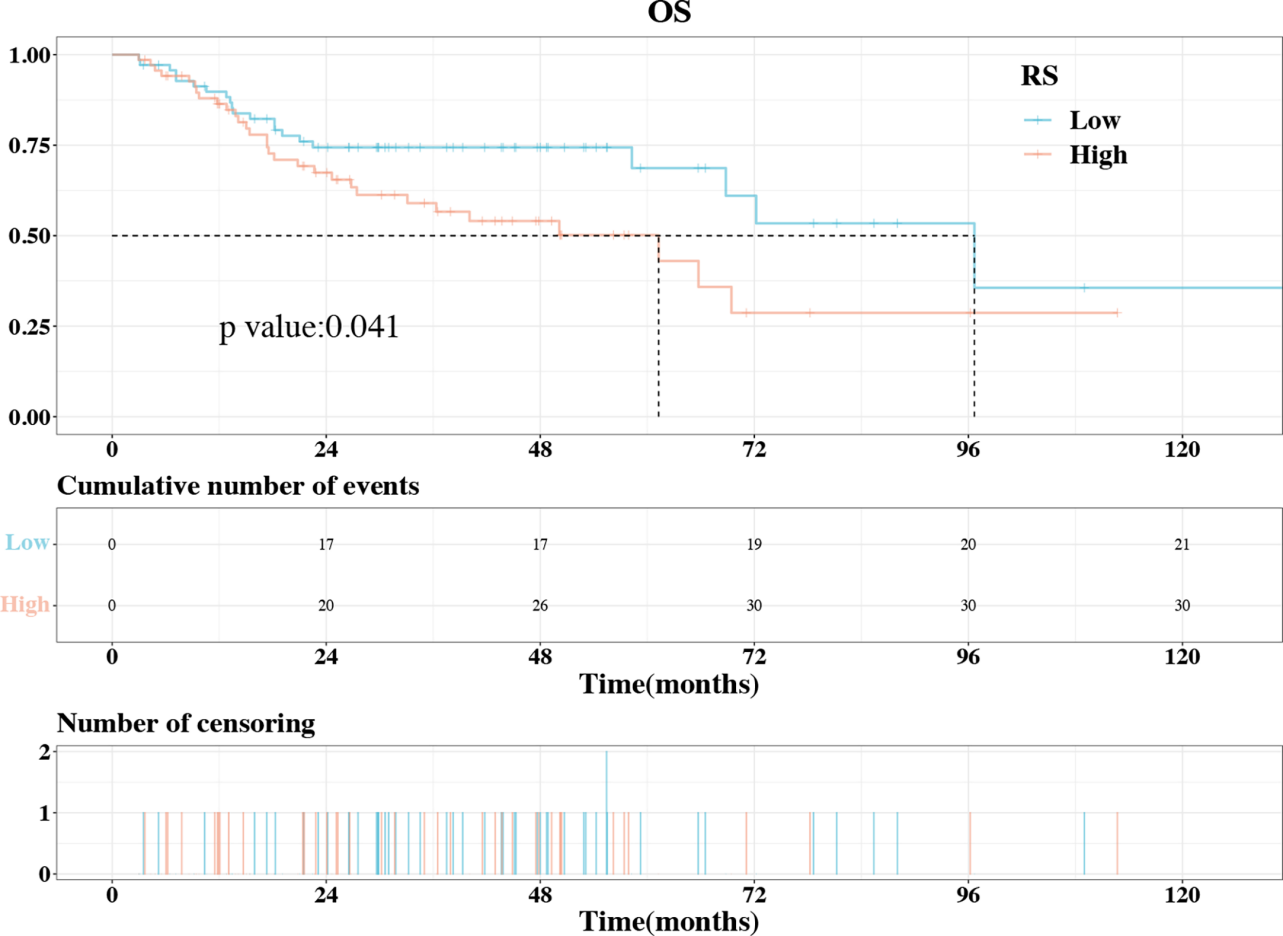
The KM plot of OS in the high rad-score and low rad-score groups. The OS was significantly higher in the low rad-score group compared to high rad-score group. KM, Kaplan–Meier; OS, overall survival.

The IL1B expression level was strongly associated with the prognosis of HNSCC and was well-predicted by the radiomics model. As a result, we used KM curves to investigate the association between the rad-score and OS in patients with HNSCC. The optimal rad-score cutoff value was determined as 0.445. Accordingly, the 139 patients were divided into a high rad-score group (n = 69) and a low rad-score group (n = 70). According to the log-rank test, the OS of the two groups was significantly different (*P* = 0.041, **Figure 9**); the median OS of the high and low rad-score groups was 61.267 and 96.667 months, respectively. The survival rates of the high and low rad-score groups at 65.7 months were 35.859% (95%CI:20.667%-62.194%) and 68.668% (95%CI:55.584%-84.832%), respectively. The corresponding survival rates at 96.5 months were 28.682% (95%CI:14.187%-57.985%) and 35.606% (95%CI:14.501%-87.424%), respectively. KM survival analysis indicated that a higher rad-score resulted in worse OS.

## 4 Discussion

The biological and location heterogeneity of HNSCC makes it difficult to select the best treatment strategy. IL1B is a vital inflammatory cytokine that significantly affects the HNSCC bioprocess. We hypothesized that the IL1B features driving a new tumor subtype that could be reflected in their OS time and could be predicted using the radiomics method. In this study, we: 1) identified the prognostic value and biological process of IL1B in HNSCC; 2) performed unsupervised analysis of radiomics features and constructed and validated a CECT-based radiomics model to predict IL1B expression; and 3) evaluated the feasibility of a CECT-based radiomics model for predicting the prognosis of patients with HNSCC. Based on these findings, we believe that non-invasive prediction of IL1B expression using a CECT-based radiomics model is helpful for personalized treatment of patients with HNSCC.

Although debatable, several studies have shown that high IL1B expression is strongly associated with poor prognosis in HNSCC (29). Our study also demonstrated that higher expression of IL1B was linked to poorer survival rate through KM analysis, and high IL1B expression was identified as a predictor for shorter OS in patients with HNSCC based on Cox regression analysis. Moreover, subgroup analysis suggested that a greater survival benefit from low IL1B expression was experienced by subgroups with radiotherapy and chemotherapy combined with surgery therapy in patients with HNSCC.

With the advancement of radiomics technology, a growing number of vital molecular markers and crucial marker cells have been predicted accurately. In contrast to histological techniques, which require intrusive sampling and only represent a small portion of the tumor, radiomic features are acquired by non-invasive preoperative examination, are easily repeated over time, and reveal the characteristics of the complete tumor. Zheng et al. (30) applied CECT-based radiomic features to predict PD-L1 expression in HNSCC; the model yielded AUCs of 0.852 and 0.802 in the training and validation cohorts, respectively. Wang et al. (31) used a multiparametric CECT radiomics model to predict the infiltration status of CD8+ T cells in patients with HNSCC; the radiomics model achieved an AUC of 0.786. In the current study, based on mRMR and REF feature selection and GBM analysis, the prediction performance of the radiomics model in the training and validation cohorts was satisfactory, yielding AUCs of 0.861 and 0.703, respectively. It should be noted that this radiomics model predicts IL1B expression in tumor tissue rather than serum IL1B. The expression of IL1B in tumor tissue is more closely related to the prognosis of HNSCC, while serum IL1B is also altered by non-tumor inflammatory diseases, such as periodontitis, arthritis, and enteritis, among others.

What makes the IL1B different from the other biomarker, is that IL1B can be regulated by canakinumab, a kind of IL1B inhibitory antibody that has been validated in several clinical trials. In the absence of histological examination, patients with HNSCC can be stratified using our radiomics model, and those with high predicted IL1B expression can be selected for canakinumab adjuvant therapy. Although no clinical trials have reported the use of IL1B-related monoclonal antibodies as adjuvant therapy in HNSCC treatment, they have been utilized in trials for other oncological disorders. Canakinumab reduced inflammatory markers and the occurrence of adverse events in pediatric patients with sickle cell anemia (32). Furthermore, canakinumab has been demonstrated to significantly reduce lung cancer incidence and mortality (20, 33). Canakinumab has also been used in the treatment of Covid-19 (34).

To the best of our knowledge, this study is the first to construct a radiomics model for predicting IL1B expression in patients with HNSCC. The radiomics model consists of three features: kurtosis from the first-order features, sphericity from the shape features, and small area emphasis (SAE) from the glszm features. Among these features, kurtosis is a measure of the peakiness of intensity distribution in an area of interest image. A previous study confirmed that kurtosis was significantly higher in HPV+ oropharyngeal squamous cell carcinoma (35). Kurtosis showed a significant difference between well- and poorly- differentiated oral squamous cell carcinoma (36). In our study, kurtosis was also proved to be an important indicator of IL1B expression. Another significant morphological feature is the 3D shape feature sphericity. Sphericity of a tumor area is a measure of its roundness compared to a sphere. Multiple radiomic investigations have demonstrated that sphericity of the primary tumor and homogeneity of the lymph nodes are predictors of pathological response (37). Davey et al. reported that sphericity is strongly associated with OS, and low sphericity is associated with worse prognosis (38). The last component of our radiomics model is the SAE, which is a measure of the distribution of small size zones, with a higher value indicating a larger number of small size zones and finer textures. Zhang et al. (39) reported that radiomics features, including SAE, could help diagnose lymph node metastasis in cervical cancer. The radiomics model built on these features allows non-invasive and effective screening of patients who require anti-IL1B adjuvant therapy. Thus, the prognosis of patients with HNSCC can be improved in a targeted manner.

Despite the fact that the radiomics model functions well, the inadequacies of this study may indicate suggestions for further research. First, clinical characteristics, such as tumor grade and TNM staging, were not combined with the radiomics model to construct a radiomics nomogram. Combining clinical characteristics with radiomics features might improve prognostic prediction accuracy. Second, all data were obtained from public sources, which may result in bias in the image quality of CECT. Third, although 139 patients were enrolled in this retrospective investigation, the robustness and reproducibility of the radiomics model may be further enhanced by a larger sample size and prospective research strategy. Fourth, the lack of external validation is a shortcoming of this study. We are actively promoting carrying out a prospective radiomics research to validate our results.

## 5 Conclusions

In conclusion, this study identified the underlying function and prognostic value of IL1B in HNSCC, constructed and validated a radiomics model based on CECT to predict IL1B expression to support individualized treatment, and evaluated the feasibility of the radiomics model for predicting the prognosis of patients with HNSCC.

## Data availability statement

Publicly available datasets were analyzed in this study. This data can be found here: The RNA-Seq data can be found in the TCGA database [http://portal.gdc.cancer.gov/] and the University of California Santa Cruz Xena platform [http://xena.ucsc.edu/]; The CECT data can be found in the TCIA database [https://www.cancerimagingarchive.net/].

## Author contributions

Conceptualization, YX, MW, HX, HS, YY, JJ and LC; Funding acquisition, LC; Investigation, YX, MW, HX, HS, YY, JJ and LC; Methodology, YX, MW, HX, HS, YY and LC; Project administration, LC; Software, YX, MW, HX, HS, YY and LC; Validation, YX, MW, HX, HS and LC; Writing – original draft, YX and MW; Writing – review & editing, LC. All authors contributed to the article and approved the submitted version.

## Supplementary material

Supplementary Table 1 The 107 radiomic features extracted with pyradiomics and their intraclass correlation coefficients.

Supplementary Table 2 Clinicopathological characteristics and IL1B expression of HNSCC tissues in training and validation cohorts.

Supplementary Table 3 The 20 radiomic features selected using mRMR algorithm. mRMR, maximum-relevance minimum-redundancy.

Supplementary Table 4 The three radiomic features and their importance in the radiomics model.

Supplementary Table 5 The list of EMT-related genes. EMT, epithelial-to-mesenchymal transition.
